# Resveratrol Plays a Protective Role against Premature Ovarian Failure and Prompts Female Germline Stem Cell Survival

**DOI:** 10.3390/ijms20143605

**Published:** 2019-07-23

**Authors:** Yu Jiang, Zhaoyuan Zhang, Lijun Cha, Lili Li, Dantian Zhu, Zhi Fang, Zhiqiang He, Jian Huang, Zezheng Pan

**Affiliations:** 1 Medical College, Nanchang University, Nanchang 330006, China; 2 Fuzhou Medical College of Nanchang University, Nanchang 344000, China; 3 The Key Laboratory of Reproductive Physiology and Pathology of Jiangxi Provincial, Nanchang University, Nanchang 330031, China; 4 Faculty of Basic Medical Science, Nanchang University, Nanchang 330006, China

**Keywords:** premature ovarian failure, resveratrol, oxidative stress, inflammation, female germline stem cells, hedgehog

## Abstract

This study was designed to investigate the protective effect of resveratrol (RES) on premature ovarian failure (POF) and the proliferation of female germline stem cells (FGSCs) at the tissue and cell levels. POF mice were lavaged with RES, and POF ovaries were co-cultured with RES and/or GANT61 in vitro. FGSCs were pretreated with Busulfan and RES and/or GANT61 and co-cultured with M1 macrophages, which were pretreated with RES. The weights of mice and their ovaries, as well as their follicle number, were measured. Ovarian function, antioxidative stress, inflammation, and FGSCs survival were evaluated. RES significantly increased the weights of POF mice and their ovaries as well as the number of follicles, while it decreased the atresia rate of follicles. Higher levels of Mvh, Oct4, SOD2, GPx, and CAT were detected after treatment with RES in vivo and in vitro. RES treatment resulted in significantly lower TNF-α and IL-6 concentrations and an obviously higher IL-10 concentration in the ovaries. In FGSCs, higher Mvh, Oct4, and SOD2 concentrations and lower TNF-α, IL-6, and MDA concentrations were measured in the RES group. Blockage of the Hh signaling pathway reversed the protective effect of RES on FGSCs. In conclusion, RES effectively improved the ovarian function of the POF model and the productive capacity of FGSCs via relieving oxidative stress and inflammation and a mechanism involving the Hh signaling pathway, suggesting that RES is a potential agent against POF and can aid in the survival of FGSCs.

## 1. Introduction

Premature ovarian failure (POF), also called premature ovarian insufficiency (POI), is defined as a loss of normal ovarian function before the age of 40 in women and is characterized by premature depletion of the ovarian follicles or folliculogenesis arrest [1]. POF is characterized by low levels of hormones and high levels of gonadotropins. It takes place in approximately 1% of women aged under 40 years and in 0.1% of women aged under 30 years [2]. Clinical symptoms include irregular amenorrhea, difficult conceiving, hot flashes, night sweats, vaginal dryness, irritability concentrating, and so on [3]. The reasons for POF include possible chromosomal defects, toxin exposure, and autoimmune disease, but in most cases, the cause remains unknown [4,5,6]. For women of reproductive age with cancer, chemotherapy and radiation damage gonadal function and are an important reason for the occurrence of POF [7,8]. Therefore, POF therapy is beneficial for the recovery of fertility and maintenance of menstrual cycles. The classic chemotherapy drugs cyclophosphamide (CTX) and busulfan (BU) are often used to construct the POF animal model based on their serious levels of reproductive toxicity [9,10].

Female germline stem cells (FSGCs) are capable of producing oocytes and replenishing the primitive follicular pool for the protection of fertility function of women undergoing chemotherapy [11,12]. Hence, the maintenance of FGSC reproductive capacity exerts a protective effect against POF and provides a new clinical research direction. Unfortunately, the mechanism of FGSC proliferation regulation is rarely reported. Stem cell self-renewal and differentiation are strictly regulated by homeostasis of the surrounding microenvironment or niche, especially the level of oxidative stress and inflammation in the microenvironment [13,14,15]. Guo et al. [16] cultured embryonic stem cells (ESCs) in vitro and found that an increase in reactive oxygen species (ROS) could induce short-term arrest of the G2/M cell cycle, indicating that the level of oxidative stress can inhibit the proliferation of ESCs. Tumor necrosis factor α (TNF-α) can inhibit the differentiation of mesenchymal stem cells (MSCs) into osteoblasts through the ubiquitin E3 ligase Wwp1 [17], and increased levels of IFN-γ and TNF-α synergistically damage the MSCs in mice [18], indicating that oxidative stress and an inflammatory environment impact the survival and proliferation of stem cells. The above research indicates that oxidative stress and the inflammation reaction may participate in the regulation of FGSCs proliferation or survival.

Resveratrol (RES) is a kind of natural phytoalexin that is widely abundant in grapes, fruit mulberry, and other plants [19]. It can mediate a number of pharmacological effects including anti-tumor [20], immune regulation, anti-inflammatory [21,22], anti-oxidation, cell protection [23,24], and anti-apoptosis [25] effects. Follicle dysfunction is related to ovarian inflammation [26], and the accumulation of ROS can intensify the process of aging [27]. RES can effectively clear ROS accumulation [28]. Follicular cells were shown to be free from cisplatin oxidative toxicity following the removal of excess ROS in Sprague-Dawley mouse ovaries [29]. In addition, RES also can improve the structure of ovarian follicles via reducing LH, the ratio of LH/FSH, and the TNF-α level [30]. The above studies suggest that RES may play a role in the improvement of premature ovarian aging through improving the environment of follicles and FGSCs, thereby promoting their survival.

Here, the activities of the antioxidative enzymes glutathione peroxidase (GPx), superoxide dismutase (SOD), and catalase (CAT) and the contents of the small molecules glutathione (GSH), ROS, and malondialdehyde (MDA) were used to assess oxidative stress levels in tissues or cells [31,32,33]. TNF-α, interleukin (IL)-6, and IL-10 were used to evaluate the degree of inflammation. Mouse Vasa Homologue (*Mvh*), an ATP-dependent RNA helicase, is specifically expressed at various stages of the germ cell life cycle and is considered to be the germline cell-specific marker gene [34,35,36]. *Oct4/Pou5f1*, which belongs to the POU domain family of transcription factors, is expressed in stem cells and is considered a stem-cell-specific marker gene [37]. Here, expression levels of *Mvh* and *Oct4* were used to evaluate ovarian function and FGSCs reproduction.

The aim of this study was to explore the protective effects of RES on ovarian function and FGSCs survival in vivo and in vitro. In addition, the possible mechanisms of these effects were investigated by altering the level of inflammation and the antioxidative stress ability.

## 2. Results

### 2.1. Protective Function of Resveratrol on the Follicle Number and Development in a Cyclophosphamide-Induced POF Model

**Figure 1:**
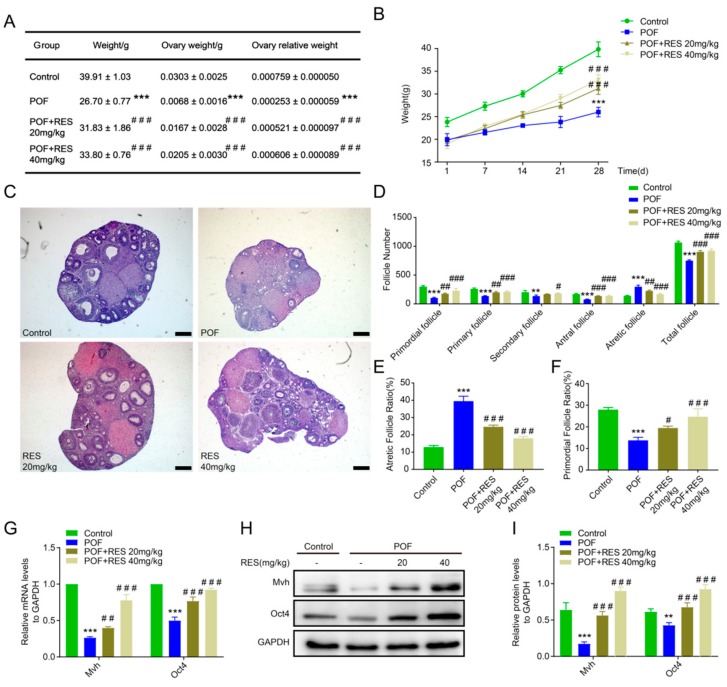
Protective function of resveratrol on follicle number and development in the cyclophosphamide-induced premature ovarian failure (POF) model. (**A**): The mouse weight, ovarian weight, and ovary/mouse relative weight ratio in control, POF, resveratrol (RES) 20mg/kg, and RES 40mg/kg groups after 28 days of continuous lavage, individually; (**B**): the total weights of mice per week in different treated groups; (**C**): the follicles were observed after HE staining, the scale bar is 200μm; (**D**): all levels of follicle number were counted by tissue slice; (**E**,**F**): the rate of follicle atresia and the primordial follicular rate vs. the total number of follicles in each group; (**G**–**I**): the mRNA and protein expression levels of *Mvh* and *Oct4* in each group. ** *p* < 0.01, and *** *p* < 0.001. vs the control group, # *p* < 0.05, ## *p*< 0.01 and ### *p* < 0.001 vs. the POF group.

Lavage was performed on the POF model mice with two doses of resveratrol (20 and 40 mg/kg) for *28* days. Then, the mouse weights, ovarian weights, and ovary/mouse relative weights were calculated, individually. The results showed that the weights of mice in the POF group were significantly lower compared to those in the control group, while mice in both the 20 and 40 mg/kg RES groups were observed to have higher weights compared to those in the POF group (*p* < 0.001, Figure 1A). We also counted the total mouse weight per week. The POF model mice gained weight slowly, but the RES group showed faster growth (*p* < 0.001, Figure 1B). Although HE staining showed that the follicle number of mice in the RES-treated groups was less than in mice with normal ovaries, all levels of follicles were observed in the RES-treated groups (20 and 40 mg/kg), but follicles were rarely detected in the POF group (Figure 1C). Counting the number of follicles in all stages showed that RES treatment elevated the proportions of primordial and primary follicles compared to the POF group (*p* < 0.001 or *p* < 0.01, Figure 1D). The number or ratio of atretic follicles dropped after RES treatment, especially in the 40mg/kg RES group, compared with the growth in the POF group (*p* < 0.001, Figure 1D,E), and the primordial follicle ratio rose gradually as the RES concentration increased (*p* < 0.001 or *p* < 0.05, Figure 1F). In addition, compared with the POF group, the expression levels of the germline or stem specific markers *Mvh* and *Oct4* in the RES groups showed an obvious recovery (*p* < 0.001 or *p* < 0.01, Figure 1G–I), suggesting that RES has a protective effect on follicle number and development in POF mice.

### 2.2. Resveratrol Treatment Relieves Oxidative Stress and Benefits Germline Cells in POF Ovaries

**Figure 2:**
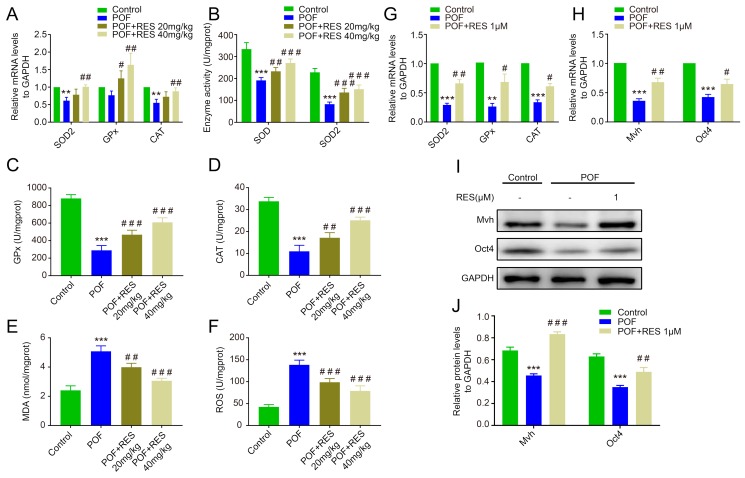
Resveratrol treatment relieves oxidative stress and benefits germline cells in POF ovaries. (**A**): The mRNA levels of superoxide dismutase 2 (SOD2), glutathione peroxidase (GPx), and catalase (CAT) in vivo; (**B**–**D**): the enzyme activity changes in total SOD, SOD2, GPx, and CAT in vivo; (**E**,**F**): the concentration alteration of malondialdehyde (MDA) and reactive oxygen species (ROS); (**G**): the mRNA levels of SOD2, GPx, and CAT; (**H**–**J**): the mRNA and protein levels of *Mvh* and *Oct4* cultured with RES in vitro. ** *p* < 0.01, and *** *p* < 0.001 vs. the control group, # *p* < 0.05, ## *p* < 0.01 and ### *p* < 0.001 vs. the POF group.

To explore the function of RES against oxidative stress in POF ovaries, we measured the mRNA level or activity of the key antioxidative enzymes in POF ovaries and the RES group. The results indicated that the expression levels of SOD2 and CAT in the POF group were down-regulated compared with the control group, while the levels of GPx, SOD2, and CAT in the 40mg/kg RES group showed obvious up-regulation compared with the POF group (*p* < 0.01, Figure 2A). The total SOD and SOD2 enzyme activity in the POF group were significantly lower than in the control group (*p* < 0.001, Figure 2C), and the activity levels of the other two key antioxidative enzymes, GPx and CAT, were also markedly decreased in the POF group compared to the control group (*p* < 0.001 or *p* < 0.01, Figure 2B,D). RES treatment resulted in significantly higher levels of total SOD, SOD2, GPx, and CAT than the levels in the POF group in a dose-dependent manner (*p* < 0.001 or *p* < 0.01, Figure 2B–D). In addition, the levels of MDA and ROS presented a dramatic up-regulation in the POF group and then dropped gradually after treatment with RES (*p* < 0.001 or *p* < 0.01, Figure 2E,F). Further, we isolated the POF ovaries and cultured them for 72 h with 1 uM RES in vitro. Corresponding to the changes in vivo, the mRNA expression levels of three antioxidative enzymes dramatically decreased in the POF group compared to those in the normal group, and RES treatment distinctly rescued the enzyme activity of POF ovaries (*p* < 0.05 or *p* < 0.01, Figure 2G). The RT-PCR and Western blot analyses showed that RES treatment partially but markedly elevated the expression levels of *Mvh* and *Oct4* compared with the POF ovaries, suggesting a rescuing contribution of RES to germline cells in the POF model (*p* < 0.05, *p* < 0.01 or *p* < 0.001, Figure 2H–J).

### 2.3. Resveratrol Enhances the Antioxidative Capacity of FGSCs and Improves Cell Survival

**Figure 3:**
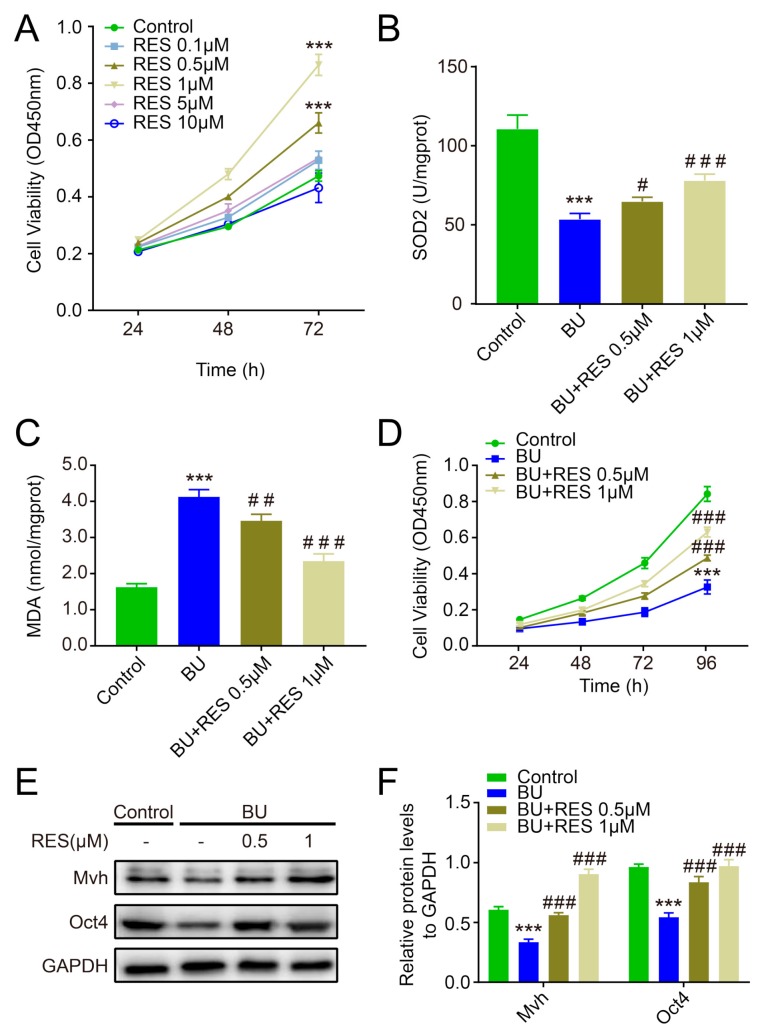
Resveratrol enhances the antioxidative capacity of female germline stem cells (FGSCs) and improves cell survival. (**A**): The CCK-8 assay was used to measure the proliferation capacity of FGSCs after co-culture with different dosages of RES; (**B**,**C**): SOD2 enzyme activity and malondialdehyde (MDA) content in different group of FGSCs; (**D**): the proliferation capacity of FGSCs (pretreated with BU) co-cultured with RES; (**E**,**F**): The protein levels of *Mvh* and *Oct4* in FGSCs of each group. *** *p*< 0.001 vs. the control group, # *p* < 0.05,## *p* < 0.01 and ### *p* < 0.001 vs. the BU group.

In order to optimize the concentration, we chose five different doses of RES to co-culture with FGSCs in the CCK-8 assay: 0.1, 0.5, 1, 5, and 10 μM. As shown in Figure 3A, compared to the normal group, the viability of FGSCs was slightly enhanced in the 0.1 and 5 μM groups, significantly increased in the 0.5 and 1 μM groups, and decreased in the 10 μM group, suggesting that 0.5 μM and 1 μM RES are suitable concentrations for FGSC proliferation (*p* < 0.001, Figure 3A). To investigate the protective effect of RES on FGSCs, RES was co-cultured with FGSCs which had been pretreated with 40 μM BU for 48h to imitate the POF model in vitro. RES treatment for 72 h resulted in a slightly higher SOD2 level in the 0.5 μM group and an obvious increase in the SOD2 level in the 1 μM group than in the BU group (*p* < 0.001 or *p* < 0.05, Figure 3B). The MDA concentration was remarkedly lower in the RES group compared to the BU group, whereas it was higher than in the normal group (*p* < 0.001 or *p* < 0.01, Figure 3C). Although FGSC proliferation was significantly inhibited by BU, RES effectively improved the viability of FGSCs in the CCK-8 analysis (*p* < 0.001, Figure 3D). Western blotting experiments showed that Mvh and Oct4 expression levels were significantly down-regulated in the BU group, and RES treatment for 72h reversed this change (*p* < 0.001, Figure 3E,F).

### 2.4. Effects of Resveratrol against Abnormal Inflammation in POF Mice

**Figure 4:**
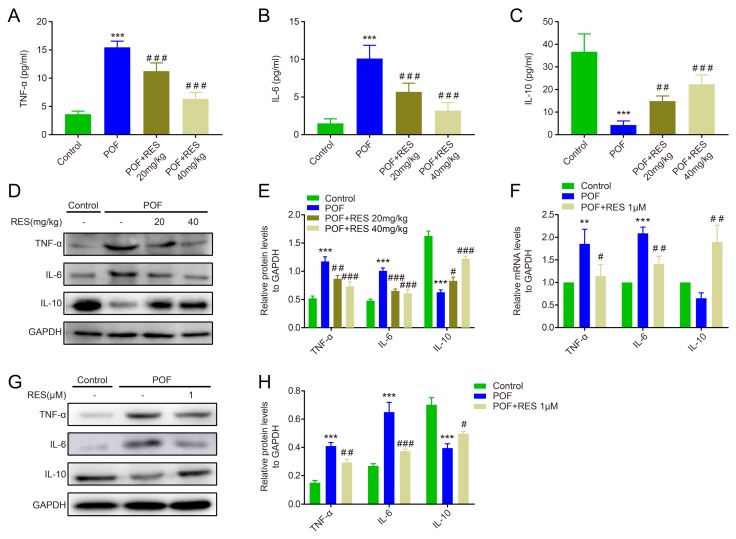
The effects of resveratrol against abnormal inflammation in POF mice. (**A**–**C**): The ELISA assay detected the levels of TNF-α, IL-6, and IL-10 in serum, respectively; (**D**,**E**): the protein levels of TNF-α, IL-6, and IL-10 in the ovaries, measured in vivo; (**F**): the mRNA expression of TNF-α, IL-6, and IL-10 in the ovaries, measured in vitro; (**G**,**H**): The protein expression of TNF-α, IL-6, and IL-10 in the ovaries, measured in vitro. ** *p* < 0.01, and *** *p*< 0.001 vs. the control group, # *p* < 0.05, ## *p* < 0.01, and ### *p* < 0.001 vs. the POF group.

After continuous lavage for 28 days with RES, the serum of POF mice was collected to measure the levels of inflammatory factors such as TNF-α, IL-6, and IL-10. The key pro-inflammatory factors TNF-α and IL-6 were significantly down-regulated in both low-dose (20 mg/kg) and high-dose (40 mg/kg) RES groups compared to the POF group in the ELISA assay, whereas the anti-inflammatory factor IL-10 was dramatically up-regulated in the RES group compared with the POF group (*p* < 0.001 or *p* < 0.01, Figure 4A–C). Corresponding to the serum data, the RES groups mediated obvious decreases in TNF-α and IL-6 protein expression in the ovaries compared with the POF ovaries, whereas they showed a marked increase in IL-10 protein expression (*p* < 0.05, *p* < 0.01, or *p* < 0.001, Figure 4D,E). In addition, the POF ovaries were isolated and co-cultured with RES for 7 days in vitro. As depicted in Figure 4F, the mRNA levels of TNF-α and IL-6 were significantly decreased after treatment with 1 μM RES, while IL-10 expression was remarkably elevated in this experiment. The same trend was detected in protein expression levels (*p* < 0.05, *p* < 0.01, or *p* < 0.001, Figure 4G,H).

### 2.5. Resveratrol Improves the Inflammatory Reaction of M1 Macrophages and Promotes FGSC Survival

**Figure 5:**
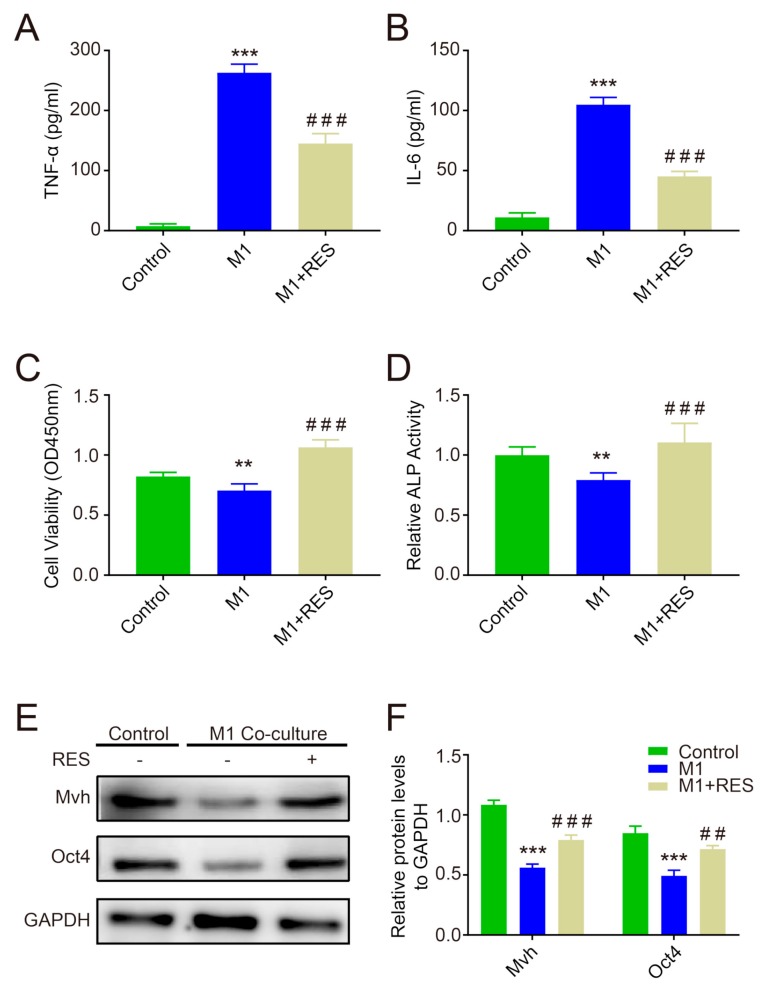
Resveratrol improves the inflammatory reaction of M1 macrophages and promotes FGSC survival. (**A**,**B**): The ELISA assay measured the TNF-α and IL-6 levels in the supernatant of the co-culture of M1 and FGSCs; (**C**) FGSC viability detection by CCK-8; (**D**): ALP activity detection; (**E**,**F**): the changes in Mvh and Oct4 protein levels. ** *p* < 0.01, and *** *p* < 0.001 vs. the control group, ## *p* < 0.01, and ### *p* < 0.001 vs. the M1 group.

After pretreatment with 20 μM RES for 48 h, M1-type macrophages (from RAW264.7) were co-cultured with FGSCs in a Transwell chamber for 48 h. The concentrations of TNF-α and IL-6 in medium were significantly decreased in the M1+RES group compared to in the M1 group, whereas they were still higher than in the control group (*p* < 0.001, Figure 5A,B). The CCK-8 assay showed that FGSCs viability was lower in the M1 group than in the control group, while it significantly increased in the M1+RES group compared to in the M1 group (*p* < 0.001 or *p* < 0.01, Figure 5C), and the ALP activity experiment also observed similar results (*p* < 0.001 or *p* < 0.01, Figure 5D). Additionally, the protein levels of Mvh and Oct4 decreased when FGSCs were co-cultured with M1 cells, whereas RES treatment obviously rescued the effect of M1 cells (*p* < 0.001 or *p* < 0.01, Figure 5E,F).

### 2.6. Blocking Hedgehog Pathway Activity Reverses the Protective Effect of Resveratrol on Ovarian Function and FGSC Survival

**Figure 6:**
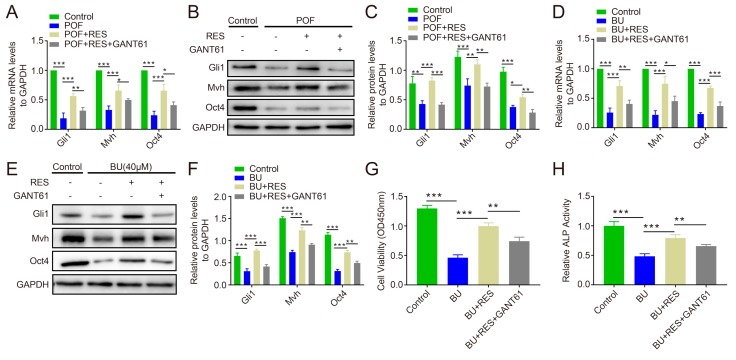
Blockage of Hh signaling activity reversed the effects of RES on ovarian function and FGSC survival. (**A**): Relative mRNA levels of Gli1, Mvh, and Oct4 in ovary tissue; (**B**,**C**): relative protein levels of Gli1, Mvh, and Oct4 at the tissue level; (**D**): relative mRNA levels of Gli1, Mvh, and Oct4 in FGSCs; (**E**,**F**): relative protein levels of Gli1, Mvh, and Oct4 in FGSCs; (**G**): detection of cell viability with the CCK-8 assay; (**H**): measurement of ALP activity in FGSCs. * *p* < 0.05, ** *p* < 0.01, and *** *p* < 0.001.

To investigate whether the Hh pathway is associated with the protective effect of RES on follicles and FGSCs, POF ovaries were isolated and treated with 1 μM RES and 1 μM RES + 10 μM GANT61 for 72 h, respectively. The results showed that the mRNA and protein levels of Gli1 were significantly higher in the RES group than in the RES + GANT61 group as well as in the POF group (*p* < 0.001 or *p* < 0.01, Figure 6A–C). Interestingly, the Mvh and Oct4 expression levels were decreased in the RES+GNAT61 group compared to in the RES group (*p* < 0.001, *p* < 0.01, or *p* < 0.05, Figure 6A–C). In FGSCs, the Gli1, Mvh, and Oct4 expression levels showed similar trends to the tissue levels (*p* < 0.001, *p* < 0.01, or *p* < 0.05, Figure 6D–F), and the CCK-8 assay showed that the viability of FGSCs was worse in the RES + GANT61 group than in the RES group (*p* < 0.001 or *p* < 0.01, Figure 6G). Similar results were also observed for the ALP activity assays (*p* < 0.001 or *p* < 0.01, Figure 6H).

## 3. Discussion

Ovarian dysfunction such as POF and premature menopause is an emerging problem for the treatment of female reproduction. The development of alternative therapeutic agents is crucial for the resumption of ovarian function [38]. RES is a polyphenolic complex that is known to have different effects under physiological and pathological conditions in various tissues and cells [39]. In cancer cells, RES has a powerful anti-inflammatory effect and antioxidative role and has been found to induce cellular apoptosis and cell-cycle arrest [40,41,42]. Bezerra et al. [43] discovered that RES promotes primordial follicle activation by reducing DNA fragmentation and stimulates granulosa cell proliferation through activation of the PI3K pathway. Li et al. [44] showed that RES reduces oxidative stress and inhibits apoptosis in granulosa cells by activating the PI3K/AKT signaling pathway in a rat model of POI.

In this study, the protective effects of RES were investigated in a mouse POF model induced by CTX/BU. We found that the weights of mice and ovaries and the ovary/mouse relative ratio obviously increased after lavaging with RES. Although the effective follicle number in the RES group was less than that in the normal group, all levels of follicles increased obviously after treatment with RES, suggesting that RES is effective in weight loss prevention and follicle protection. Meanwhile, the expression levels of *Mvh* and *Oct4* increased in the RES group, which indicated that RES partially and significantly prompted the proliferation of germline stem cells in the POF model. In our experiments, Kunming mice were chosen to construct the POF models in view of their excellent reproductive capacity and adaptability. Previous studies [45,46] have shown that FGSCs not only present in the ovaries of Kunming mice, but also exist in CD-1 and C57 mice. We believe RES is also as effective in other strains.

Although the oxidative stress markers SOD2 and CAT significantly decreased following treatment with MDA in a chemotherapy-induced mouse model [47], the significant differences in SOD and MDA in rat groups treated with cisplatin alone and RES were not observed in ovarian levels [29]. Our data showed that reduced mRNA levels of SOD2 and CAT were measured in an animal group of POF. All of the antioxidative enzyme activities were increased after treatment with RES in vivo, and the concentrations of MDA and ROS were reversed by RES. It is interesting to note that the same effects were seen in in vitro experiments. Hence, our study shows that RES has an important role in reducing oxidative stress and rescuing ovary function in a POF model induced by CTX/BU.

To investigate the protective effects of RES against oxidative stress in FGSCs, in this experiment, FGSCs were primarily cultured and treated with different concentrations of RES. The CCK-8 assay results showed that FGSCs grew well under treatment with 0.5 μM and 1 μM RES; therefore, we chose these two concentrations for the following experiments. Because CTX is a precursor drug, it needs to be activated in vivo, so 40 μM of BU was used to pretreat FGSCs to imitate the POF model in in vitro experiments. In this study, intervention with RES was shown to play a role in increasing both SOD2 activity and cellular viability with a significant decrease in MDA, indicating that RES improves the antioxidative capacity of FGSCs and protects cells against BU damage. Meanwhile, the protein levels of Mvh and Oct4 increased following treatment with RES, suggesting a protective effect of RES against chemotherapeutic injury, thereby increasing FGSC survival.

Additionally, in this study, RES was also used to evaluate the anti-inflammatory effect in the POF model. We measured the relative factors in the serum of animal models. The key pro-inflammatory factors TNF-α and IL-6 were significantly down-regulated in RES group, whereas the anti-inflammatory factor IL-10 was dramatically up-regulated compared to in the POF group. Next, lavage ovaries were isolated to measure the expression levels of these factors, and POF ovaries were co-cultured with RES in vitro. The protein levels of TNF-α, IL-6, and IL-10 both exhibited a similar trend in serum both in vivo and in vitro. The above data prove that RES can effectively reduce the level of abnormal inflammation in POF mice induced with CTX/BU.

Further, to investigate the anti-inflammatory effects and avoid the direct influence of RES on FGSCs, M1-type cells originating from the RAW264.7 macrophage were pretreated with RES for 48 h. To select the optimum concentration, we used different dosages of RES to co-culture with M1 macrophages: 5, 10, 15, 20, and 25 μM. Finally, 20 μM RES was selected as the pretreated concentration to maximize the effect of inflammation inhibition based on M1-type cell survival at this dosage (the data are not provided). As depicted in Figure 5, the pro-inflammatory factors TNF-α and IL-6 were significantly decreased, and the viability and ALP activity of FGSCs were obviously increased in the RES group compared to the M1 group. Additionally, FGSCs in the RES group showed increased protein levels of both Mvh and Oct4. Our results indicate that RES attenuated M1-type macrophage-induced inflammation injury in FGSCs and improved the cellular survival or proliferative capacity.

Our previous studies suggest that the blockage of Hh pathway activity decreases the follicle number and FGSC proliferation, and the Hh pathway showed an obvious down-regulation in POF ovaries (article is accepted). It is unclear whether the protective effect of RES on follicles and FGSCs can be reversed through the blockage of Hh pathway activity. Gli1 is the key positive transcription factor of the Hh pathway, and its expression level represents activity in the Hh pathway [48]. GANT61, a specific inhibitor of the Hh pathway [49], was used to treat POF ovaries and FGSCs with RES in vitro. Our data showed that Gli1 was significantly lower in the RES+GANT61 group than in the RES group, indicating that GANT61 can effectively inhibit the activity of the Hh pathway, even if RES exists. The Mvh and Oct4 levels decreased in the GANT61 treated group compared to that in the RES group, suggesting that inhibition of Hh pathway activity blocked the protective effect of RES on ovary function. At the cellular level, BU was used to produce reproductive toxicity in vitro to imitate the POF model. The experimental data showed that the blockage of Hh activity decreased the protective function of RES on the reproductive capacity or survival of FGSCs.

In this experiment, the mouse age is one of the important factors affecting the activity of FGSCs. In order to avoid the age factors on the experiments, the control group and treated group mice were from the same generation. For a mouse, one of the ovaries was used to the control group, another ovary was taken as the treated group. The above method tried to avoid the influence by aging. Too much chemotherapeutic drugs may cause complete depletion of FGSCs in ovaries, we performed pre-experiments to look for a suitable dosage and timing of CTX/BU based on previous studies [50,51]. An unsuitable dosage and timing of RES can cause some serious side effects, we also performed per-experiments to optimize the treatment of RES.

In conclusion, RES effectively protects the follicle number and FGSC survival through multiple mechanisms such as antioxidative stress, relief of inflammatory reactions, and involvement in Hh signaling pathway activity. These results indicate that RES is a potential protective agent against POF that may prompt the growth of FGSCs; however, further studies are needed to illuminate the molecular mechanism of RES on ovary function and FGSC survival.

## 4. Materials and Methods

### 4.1. Animals and Treatment

The 6 weeks old Kunming mice used in the experiment were provided by the Department of Animal Science of Jiangxi College of Traditional Chinese Medicine. CTX (120 mg/kg, Sigma, St. Louis, MO, USA) and BU (30 mg/kg, Sigma, USA) were administrated a single dose by intraperitoneal injection and the mice were observed for 4 weeks to construct the POF model. RES (Sigma, USA) was dissolved in physiological saline and lavaged for 28 days. The RES treatment group was divided into a low concentration group (20 mg/kg) and high concentration group (40 mg/kg), respectively. The weights of the mice were recorded each week, and the relative weights of the ovaries (the ratio of bilateral ovarian weight to body weight) were calculated on the final day of treatment. All mice were housed at the appropriate temperature, guaranteed a 12 h dark environment each day to ensure adequate rest, and had free access to food and drinking water. Animal protocol was approved by the Animal Care Committee of Nanchang University Jiangxi Medical College (Animal protocol: NCDXSYDWFL-2015097).

### 4.2. Ovary Extraction and Culture

The ovaries were dissociated and placed in pre-cooling PBS solution and as much surrounding tissue was removed as possible. Ovaries were placed in the Transwell chamber (Millicell, Darmstadt, Germany) in Waymouth medium (Sigma, USA) containing 10% (*v*/*v*) FBS (Gibco, Staley Rd Grand Island, NY, USA), 0.23 mM sodium pyruvate, and penicillin and streptomycin (P/S) (Solarbio, Beijing, China).

### 4.3. HE Staining and Follicle Counting

The ovary tissue was fixed overnight in 4% paraformaldehyde at room temperature and then embedded in paraffin. Hematoxylin and eosin (HE) staining was used to observe the morphological characteristics of the ovaries and calculate the number of follicles at all levels. Follicle counts refer to the follicle classification criteria by Chen et al. [52], and the atresia rate (the ratio of the number of atresia follicles to the total number of follicles) and the primordial follicle rate (the ratio of the number of primordial follicles to the total number of follicles) were calculated.

### 4.4. Sample Preparation and Biochemical Analysis

After anesthesia, the eyeballs of the mice were removed to collect blood samples. After standing at room temperature to clot the blood samples for 60 min, they were centrifuged at 3000 rpm for 20 min, and the supernatant was collected for storage. The ELISA kits (Elabscience Biotechnology, Wuhan, China) were used to detect the levels of the inflammatory factors TNF-α, IL-6, and IL-10 in mouse serum or cell supernatant. All biochemical test kits were supplied by KeyGEN BioTECH (Nanjing, China). Briefly, after the end of treatment, the ovaries from both sides of each mouse was removed, and the tissue homogenate was prepared after weighing. The levels of ROS (KGT010, KeyGEN BioTECH, Nanjing, China), MDA (KGT003-1, KeyGEN BioTECH, Nanjing, China), GSH (KGT006, KeyGEN BioTECH, Nanjing, China), and the enzyme activities of GPx (KGT014, KeyGEN BioTECH, Nanjing, China), total SOD, SOD2 (KGT00150-1, KeyGEN BioTECH, Nanjing, China), and CAT (KGT017, KeyGEN BioTECH, Nanjing, China) were detected in the ovarian tissue. The treatment and reaction conditions were in accordance with the kit instructions.

### 4.5. Primary Isolation and Culture of FGSCs

The 3-day postnatal mouse ovaries were collected and placed in pre-cooled PBS buffer to remove surrounding tissues. The FGSCs were separated via the modified two-step enzymatic digestion method. In brief, the ovaries were cut into 3–4 pieces in PBS buffer without Ca^2+^ and Mg^2+^, collected by filtration, and transferred into collagenase IV (1 mg/mL, Sigma, USA). After digesting the section for 13 min at 37 °C, the solution was centrifuged at 500 g for 5 min, and the supernatant was discarded, resuspended in 0.05% trypsin (Sigma, USA) containing EDTA (1 mM, Sigma, USA), and digested for 2.5 min at 37 °C. Serum was added to stop the reaction. FGSCs were cultured in the specific medium containing minimum essential medium-α (MEM-α), 10% (*v*/*v*) FBS (Gibco, USA), 1 mM sodium pyruvate, 1 mM non-essential amino acids, 2 mM L-glutamine, 0.1 mM β-mercaptoethanol (Sigma), 20 ng/mL LIF (Sigma), 10 ng/mL mEGF (Sigma), 40 ng/mL GDNF (Sigma), 1 ng/mL bFGF (Sigma) and 100×penicillin and streptomycin (P/S) (Solarbio, Beijing, China). The culture medium was changed daily to maintain the stability of the pH and drug concentration in the culture system.

### 4.6. Cell Treatment and Proliferation Activity Assay

FGSCs were treated with different concentrations of resveratrol (0.1, 0.5, 1, 5, and 10 μM) to screen for appropriate dosing concentrations. FGSCs were treated with busulfan (40 μM) for 48 h to induce chemotherapy-induced reproductive toxicity damage. M1 macrophages (RAW 264.7) were provided by the Chinese Academy of Sciences cell bank, placed in the lower chamber of the Transwell co-culture system, and the FGSCs were located in the upper chamber. GANT61 (5 μM) was treated for 48 h to inhibit the expression level of the Hedgehog signaling pathway in FGSCs. FGSCs (2000 cells per well) were plated in 96-well plates, and a Cell Counting Kit (CCK8, Transgen BioTECH, Beijing, China) was used to detect cell proliferation activity.

### 4.7. ALP enzyme Activity Assay

An ALP enzyme activity assay kit (Solarbio, China) was used to detect the level of ALP enzyme activity in FGSCs. Briefly, the FGSCs suspension was cleaved into homogenate and then subjected to a color reaction according to the operation guidelines. After sufficient reaction time, the absorbance of the reaction solution was measured at a wavelength of 510 nm, and the relative enzyme activity level was calculated by comparison with the standard.

### 4.8. Quantitative Real-Time PCR

Trizol reagent (ER501-01, TransGen Biotech, China) was used to extract the total mRNA from tissue or cell samples. cDNA was obtained by reverse transcription according to the method of the PrimeScript RT reagent kit (R047A, TAKALA, Shiga, Japan), which was prepared for subsequent detection. TB Green Mix (R820A, TAKALA, Japan) was used for real-time quantitative PCR. The PCR primer sequences are shown in Table 1 and are only used for PCR amplification of specific fragments of interested genes. The housekeeping gene *GAPDH* was used as an internal reference. Finally, GraphPad Prism 7 software was used for statistical analysis.

### 4.9. Western Blotting

Western blotting was performed as described previously [53]. Briefly, proteins in tissue or cell samples were extracted by RIPA Lysis Buffer (Applygen, Beijing, China). Protein concentrations were measured with a BCA kit (Applygen, China). The proteins underwent polyacrylamide gel electrophoresis and were then transferred to the PVDF membrane (Millipore, Darmstadt, Germany). The membranes were blocked with 5% bovine serum albumin solution for 6 h and then the primary antibodies were incubated. The primary antibodies used in this study included Mvh (ab27591, abcam, Cambridge, UK), Oct4 (ab18976, abcam, Cambridge, UK), TNF-alpha (ab6671, abcam, Cambridge, UK), IL-6 (ab208113, abcam, Cambridge, UK), IL-10 (ab9969, abcam, Cambridge, UK), Gli1 (DF7523, Affinity Biosciences, Cincinnati, OH, USA), and GAPDH (ab181602, abcam, Cambridge, UK). After incubation of the secondary antibody (Affinity Biosciences, Cincinnati, USA), the blots were imaged using the EasySee Western Blot Kit (DW101-01, TransGen Biotech, China). Analyzer Image AI600 and Image J were used to scan and analyze the images.

### 4.10. Statistical Analysis

The statistical analysis was performed using GraphPad Prism 7 software. One-way ANOVA was used to detect statistical differences between multiple sets of data. A *p*-value < 0.05 was considered statistically significant. All data are presented as the mean ± standard error from at least three independent experiments.

## Figures and Tables

**Table 1:** Sequences used for quantitative real-time PCR.

Genes	Primer Sequence (5′-3′)	Gene Number
*Mvh*	Forward:GTGTATTATTGTAGCACCAACTCG Reverse:CACCCTTGTACTATCTGTCGAACT	NM_001145885.1
*Oct4*	Forward: AGCTGCTGAAGCAGAAGAGG Reverse: GGTTCTCATTGTTGTCGGCT	NM_013633.3
*GPx*	Forward: GGCTCATCTGAGCAACAAGG Reverse: CGCCGATGTTCAAGGTCAAT	NM_008160.6
*SOD2*	Forward: ACAACTCAGGTCGCTCTTCA Reverse: GAACCTTGGACTCCCACAGA	NM_013671.3
*CAT*	Forward: ACATGGTCTGGGACTTCTGG Reverse: ACTGCCTCTCCATCTGCATT	NM_009804.2
*TNF-α*	Forward: CTCATGCACCACCATCAAGG Reverse: ACCTGACCACTCTCCCTTTG	NM_013693.3
*IL-6*	Forward: GACTGATGCTGGTGACAACC Reverse: AGACAGGTCTGTTGGGAGTG	NM_031168.2
*IL-10*	Forward: AGTACAGCCGGGAAGACAAT Reverse: TCTAGGAGCATGTGGCTCTG	NM_010548.2
*Gli1*	Forward: CCCAATACATGCTGGTGGTG Reverse: GCAACCTTCTTGCTCACACA	NM_010296.2

## Abbreviations

POF	Premature ovarian failure
POI	Premature ovarian insufficiency
CTX	Cyclophosphamide
BU	Busulfan
FSGC	Female germline stem cell
ESC	Embryonic stem cell
ROS	Reactive oxygen species
MSC	Mesenchymal stem cells
Mvh	Mouse vasa homologue
RES	Resveratrol
GPx	Glutathione peroxidase
SOD	Superoxide dismutase
CAT	Catalase
GSH	Glutathione
MDA	Malondialdehyde
Hh	Hedgehog
TNF-α	Tumor necrosis factor α
